# Full-length soluble urokinase plasminogen activator receptor down-modulates nephrin expression in podocytes

**DOI:** 10.1038/srep13647

**Published:** 2015-09-18

**Authors:** Massimo Alfano, Paola Cinque, Guido Giusti, Silvia Proietti, Manuela Nebuloni, Silvio Danese, Silvia D’Alessio, Marco Genua, Federica Portale, Manuela Lo Porto, Pravin C. Singhal, Maria Pia Rastaldi, Moin A. Saleem, Domenico Mavilio, Joanna Mikulak

**Affiliations:** 1 Division of Oncology, Urological Research Institute URI; IRCCS Ospedale San Raffaele, Milan, Italy; 2 Department of Infectious Diseases, IRCCS Ospedale San Raffaele, Milan, Italy; 3 Depatment of Urology, Humanitas Clinical and Research Center, Rozzano, Milan, Italy; 4 Pathology Unit, L. Sacco Department of Biomedical and Clinical Sciences, University of Milan, Milan, Italy; 5 IBD Center, Humanitas Clinical and Research Center, Rozzano, Italy; 6 Unit of Clinical and Experimental Immunology, Humanitas Clinical and Research Center, Rozzano, Milan, Italy; 7 Center for Excellence for Immunology and Inflammation, Feinstein Institute for Medical Research, Hofstra North Shore Long Island Jewish Medical School, New Hyde Park, NY, USA; 8 Renal Research Laboratory, Fondazione IRCCS Ospedale Maggiore Policlinico and Fondazione D’Amico per la Ricerca sulle Malattie Renali, Milan, Italy; 9 Children's Renal Unit and Academic Renal Unit, University of Bristol, Bristol, United Kingdom; 10 Department of Medical Biotechnologies and Translational Medicine (BioMeTra), University of Milan, Milan, Italy

## Abstract

Increased plasma level of soluble urokinase-type plasminogen activator receptor (suPAR) was associated recently with focal segmental glomerulosclerosis (FSGS). In addition, different clinical studies observed increased concentration of suPAR in various glomerular diseases and in other human pathologies with nephrotic syndromes such as HIV and Hantavirus infection, diabetes and cardiovascular disorders. Here, we show that suPAR induces nephrin down-modulation in human podocytes. This phenomenon is mediated only by full-length suPAR, is time-and dose-dependent and is associated with the suppression of Wilms’ tumor 1 (WT-1) transcription factor expression. Moreover, an antagonist of αvβ3 integrin RGDfv blocked suPAR-induced suppression of nephrin. These *in vitro* data were confirmed in an *in vivo* uPAR knock out Plaur^−/−^ mice model by demonstrating that the infusion of suPAR inhibits expression of nephrin and WT-1 in podocytes and induces proteinuria. This study unveiled that interaction of full-length suPAR with αvβ3 integrin expressed on podocytes results in down-modulation of nephrin that may affect kidney functionality in different human pathologies characterized by increased concentration of suPAR.

Release of the urokinase-type plasminogen activator receptor (uPAR, encoded by *PLAUR*), is catalyzed by various enzymes and results in soluble uPAR (suPAR) that can be shed from cell membrane either in full-length suPAR (fl-suPAR) or cleaved variants (c-suPAR) thus generating different suPAR isoforms with different functions^1^,^2^,^3^,^4^,^5^,^6^,^7^. Both fl-suPAR and c-suPAR binding to its ligand uPA mediate a scavenger activity and interact with cell associated integrins in order to influence different intracellular processes^3^. Although the function(s) of c-suPAR variant is only partially known, there is evidence that this cleaved form of the protein acquires chemotactic activity by either favoring mobilization of hematopoietic stem cells from bone marrow or by inhibiting the chemotactic potential of the chemokine-enriched extracellular milieu of HIV-infected tonsils^4^,^5^,^6^.

Recently, the increased concentration of suPAR was proposed as a specific circulating risk factor for Focal Segmental Glomerusclerosis (FSGS)^8^. However, further clinical studies observed increased concentration of suPAR also in other glomerular and proteinuric disease indicating that plasma suPAR accumulation is not a specific biomarker for FSGS but increases also in other glomerular disorders characterized by decreased glomerular filtration rate (GFR) and in some cases by proteinuria^9^,^10^,^11^,^12^,^13^. Furthermore, high plasma levels of suPAR have been associated with the progression of diverse human pathologies including cancer, sepsis, liver disease, rheumatic disorders and HIV infection, sharing systemic degrees of inflammation and chronic immune activation^14^,^15^,^16^,^17^,^18^,^19^,^20^,^21^.

Podocytes are highly specialized epithelial cells that have an important function in the process of glomerular barrier filtration together with fenestrated endothelium and glomerular basement membrane. Various proteins including nephrin, synthesized by the podocytes are integral parts of the slit diaphragm and are required for the proper functioning of the renal filtration barrier. Different identified nephrin gene mutations and defects of nephrin distribution at the level of the slit diaphragm cause congenital nephritic syndromes^22^,^23^,^24^ and proteinuria^25^,^26^,^27^, respectively. Moreover, integrins in kidney play a critical role in development, homeostasis and renal diseases^28^,^29^. Recently αvβ3 integrin expressed on podocytes has been proposed as a suPAR binding molecule regulating the glomerular filtration barrier^30^,^31^,^32^,^33^. However, the mechanistic insight(s) associated to podocyte dysfunction mediated by suPAR- αvβ3 interaction have not yet been disclosed. Because of their ability to interact with cellular transmembrane receptors, full length suPAR and c-suPAR are not only mere biomarkers of disease progression, but may also be actively involved in their pathogenesis. The present study shows that both recombinant and plasma associated suPAR down-modulate nephrin at transcription and protein levels in human podocytes *in vitro*. Furthermore, we demonstrated that only the full-length form, but not c-suPAR variant, is associated with the negative modulation of nephrin through a direct interaction with αvβ3 integrin. This mechanism was further supported by an *in vivo* model of suPAR knock out (Plaur^−/−^) mouse showing that the infusion of high dose of suPAR inhibits expression of nephrin and Wilms’ tumor 1 (WT-1) in podocytes and induces proteinuria indicating that the full length suPAR might actively contribute to the podocytes dysfunction in different human pathologies.

## Results

### SuPAR induces down-modulation of nephrin in human primary podocytes

**Figure 1:**
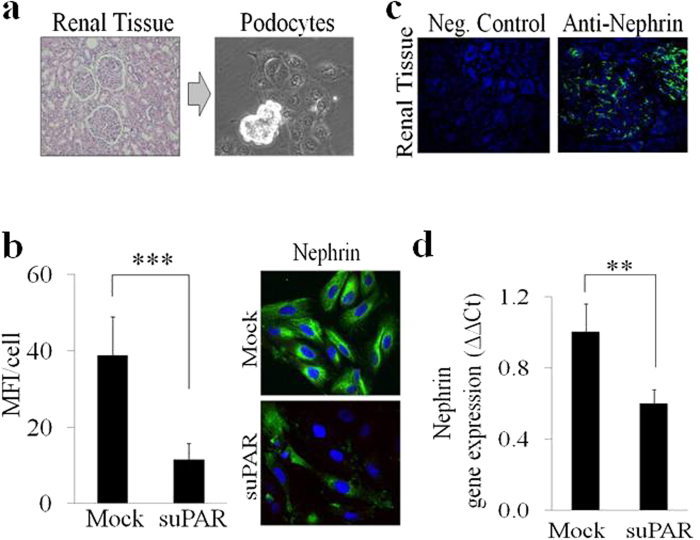
SuPAR down-modulates nephrin expression in human primary podocytes. (**a**) Haematoxylin-eosin-staining (left picture) of human kidney from patients underwent to nephrectomy due to the renal cell carcinoma. Isolated human glomerula were cultured *in vitro* in order to obtain human primary podocytes (right picture). One representative experiment out of 20 is shown. (**b**) Quantification (left panel) of the immunoflourescence staining of nephrin expression in control (Mock) and human primary isolated podocytes treated with human recombinant suPAR (20 ng/mL) for 24 hours (suPAR). Results are expressed as MFI/cell and represent the average of 3 experiments ±SD. DAPI staining was used to determine nuclei number. Right picture represent one representative experiment out of 3 of nephrin expression in green (488 Alexa Fluor) in Mock and suPAR treated human primary podocytes. A nucleus staining is show in blue (DAPI). (**c**) Immunoflourescence staining of frozen tissue from human normal kidney with the specific clonal rabbit anti-nephrin Ab (488 Alexa Fluor). (**d**) QPCR analysis of nephrin expression by using specific human TaqMan assay in Mock and suPAR treated human podocytes. Results are expressed as relative fold change in suPAR treated cells vs Mock cells (ΔΔCt) and represent the average of 3 experiments ±SD. Values were normalized to GAPDH gene expression. Statistical significance (*P*) is indicated by asterisks and is represented as: **<0.01; ***<0.001.

**Figure 2:**
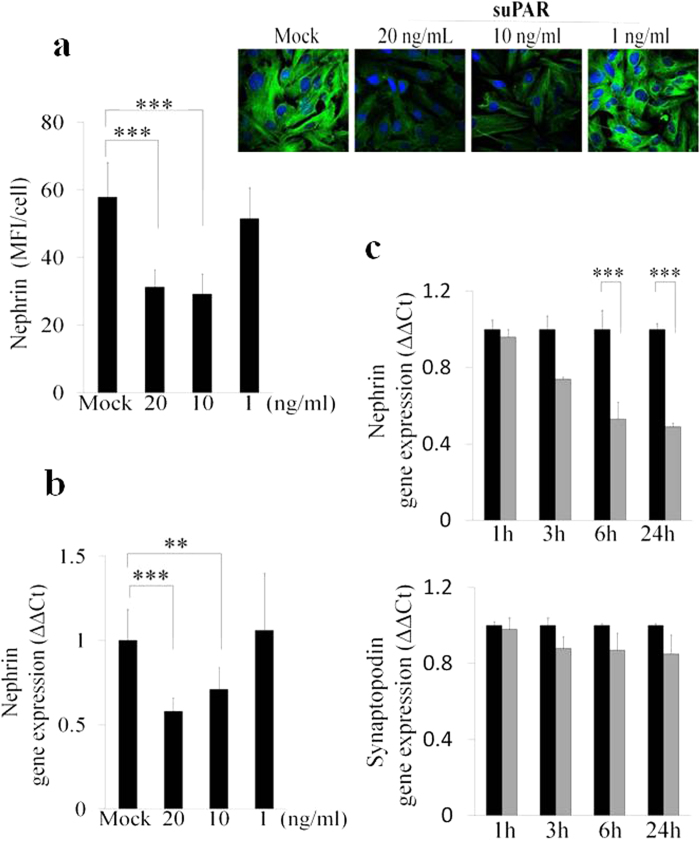
Down-modulation of nephrin expression both at protein and transcription level in CIHPs. (**a**) Quantification (left panel) of immunoflourescence staining (right upper panel) of nephrin expression in control (Mock) and CIHPs treated with different human recombinant suPAR for 24 hours (suPAR). Results are expressed as MFI/cell and represent the average of 6 experiments ±SD. DAPI staining was used to determine nuclei number. Results are expressed as MFI/cell and represent the average of 4 experiments ±SD. Right picture shows one representative immunoflourescence staining out of 4 of nephrin expression (488 Alexa Fluor) in green and nucleus (DAPI) in blue. (**b**) Dose-dependent qPCR analysis of nephrin expression in Mock and suPAR treated human podocytes by using specific human TaqMan assays. Results are expressed as relative fold change in suPAR treated cells vs Mock cells (ΔΔCt) and represent the average of 6 experiments ±SD. Values were normalized to the expression of GAPDH gene. (**c**) Time course qPCR analysis of nephrin and synaptopodin expression in Mock and suPAR treated human podocytes by using specific human TaqMan assays. Results are expressed as relative fold change in suPAR treated cells vs Mock cells (ΔΔCt) and represent the average of 6 experiments ±SD. Values were normalized to the expression of GAPDH gene. Statistical significance (*P*) is indicated by asterisks and is represented as: ***<0.001.

Various, genetic and functional, nephrin defects lead to nephritic syndromes and proteinuria^22^,^23^,^24^,^25^,^26^,^27^. To verify the effect of suPAR stimulation on nephrin expression, we used human primary podocytes obtained from renal tissue of patients affected by renal adenocarcinoma who underwent a radical nephrectomy. All tissue specimens used for this study were collected from the distal part of the pathological tissue and were free from any disease as was verified by haematoxylin-eosin-staining (Fig. 1a). We then cultured freshly isolated glomerula in order to obtain human podocytes (Fig. 1a), whose phenotype was verified by qPCR analysis detecting gene expression of the following specific podocytes markers: Wilms’ tumor 1 (WT-1), synaptopodin, nephrin and podocin (data not shown). Mean serum and plasma concentration of suPAR in healthy adults have been reported to be 2 ng/mL^18^, whereas it reaches 20 ng/mL in patients with various diseases, such as cancer, sepsis, liver disease, HIV infection and FSGS^8^,^14^,^18^,^19^,^20^,^21^. We then proceeded to incubate human primary podocytes with 20 ng/mL of human recombinant fl-suPAR. After 24 hours of stimulation we detected by fluorescence microscopy a significant down-modulation of nephrin at the protein level (Fig. 1b). Immunoflourescence staining of frozen tissue from human normal kidney served to confirm the specificity of anti-nephrin rabbit clonal antibody (Ab) used for this study (Fig. 1c). The decreased amount of nephrin following incubation of primary podocytes with suPAR was also confirmed by qPCR, thus suggesting a transcriptional control of nephrin expression by suPAR (Fig. 1d). To both confirm the modulation of nephrin exerted by suPAR and to disclose the related mechanism in a post-mitotic podocyte, we repeated the same experimental approach in conditional immortalized human podocytes (CIHPs) *in vitro*^34^. By performing assessments of protein expression with immune-flourescence in CIHPs incubated with different concentrations of suPAR for 24 hours, we detected a significant reduction of nephrin flourescence intensity and thus a lower level of nephrin protein in suPAR stimulated CIHPs comparing to control experiments, with the maximum inhibition between 10–20 ng/mL of suPAR (Fig. 2a). To determine whether suPAR stimulation affects nephrin expression at the transcriptional level, we performed qPCR experiments. As shown in Fig. 2b, quantification of nephrin mRNA in CIHPs incubated with suPAR showed a rapid and progressive decrease of *Nephrin* gene expression (Fig. 2a–c). The reduction of nephrin transcripts was already detectable after 3 hours and became significant after 6 hours of treatment, when it also reached a plateau that was maintained even after 24 hours of stimulation. However, we did not observe any significant suPAR-mediated down-modulation of the expression of synaptopodin, another specific podocyte marker, thus indicating the specificity of suPAR in suppressing nephrin expression in CIHPs (Fig. 2c).

### Full-length suPAR, but not the truncated variant, down-regulates nephrin expression in human podocytes

**Figure 3:**
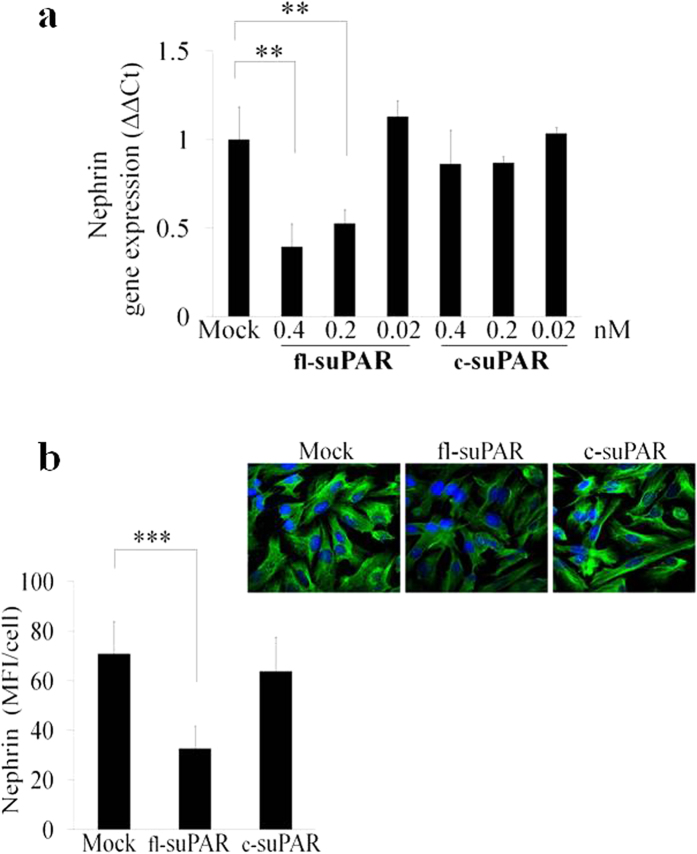
Full length D_I_D_II_D_III_ suPAR down-regulates nephrin expression in CIHPs. (**a**) QPCR analysis using specific human nephrin TaqMan assay in Mock and treated human podocytes with different concentration of full lengh suPAR (fl-suPAR) or short suPAR (c-suPAR) variants of suPAR. Results are expressed as relative fold change in suPAR treated cells vs Mock cells (ΔΔCt) and normalized to the expression of GAPDH gene. Results are represent as the average of 3 independent experiments ±SD. (**b**) Quantification of immunoflourescence staining (left panel) of nephrin expression in control (Mock) and treated human podocytes for 24 hours with 0.4 nM of the full-length (fl-suPAR) or cleaved (c-suPAR) variants of suPAR. Results are expressed as MFI/cell and represent the average of 3 experiments ±SD. Right picture shows one representative immunoflourescence staining out of 3 of nephrin expression (488 Alexa Fluor) in green and nucleus (DAPI) in blue.Statistical significance (*P*) is indicated by asterisks and is represented as: **<0.01; ***<0.001.

Structurally, uPAR is a GPI-anchored membrane glycoprotein consisting of three homologous domains (D_I_D_II_D_III_)^35^. Cleavage of uPAR from the cell membrane is catalyzed by various enzymes and can occur both at the GPI-anchor and the linker region between D_I_ and D_II_. The suPAR resulting from cleavage may thus consist of domain D_I_D_II_D_III_, D_II_D_III_ or D_I_. Since the increased level of plasma-associated suPAR in human disorders reflects the increased concentration of both D_I_D_II_D_III_ (fl-suPAR) and D_II_D_III_ (c-suPAR) variants, we evaluated the contribution of suPAR variants in down-modulation of nephrin in CIHPs^4^,^6^. To compare the dose-dependent effects of fl-suPAR and c-suPAR variants on nephrin inhibition, two suPAR variants were tested in the range of concentration between 0.4–0.02 nM that resulted with the previously observed maximum inhibitory effect of fl-suPAR (Fig. 2b). Interestingly, only fl-suPAR protein was able to significantly reduce nephrin expression at transcription level (Fig. 3a). In addition immunoflourescence analysis showed a statistically significant reduction of nephrin also at protein level only after stimulation with fl-suPAR and not with its short variant c-suPAR (Fig. 3b). These data provide strong evidences of fl-suPAR and not c-suPAR variant capability to induce nephrin down-regulation in human podocytes.

### SuPAR mediated down-regulation of nephrin in human podocytes occurs through interaction with αvβ3 integrin and is associated with suppression of the WT-1 transcription factor

**Figure 4:**
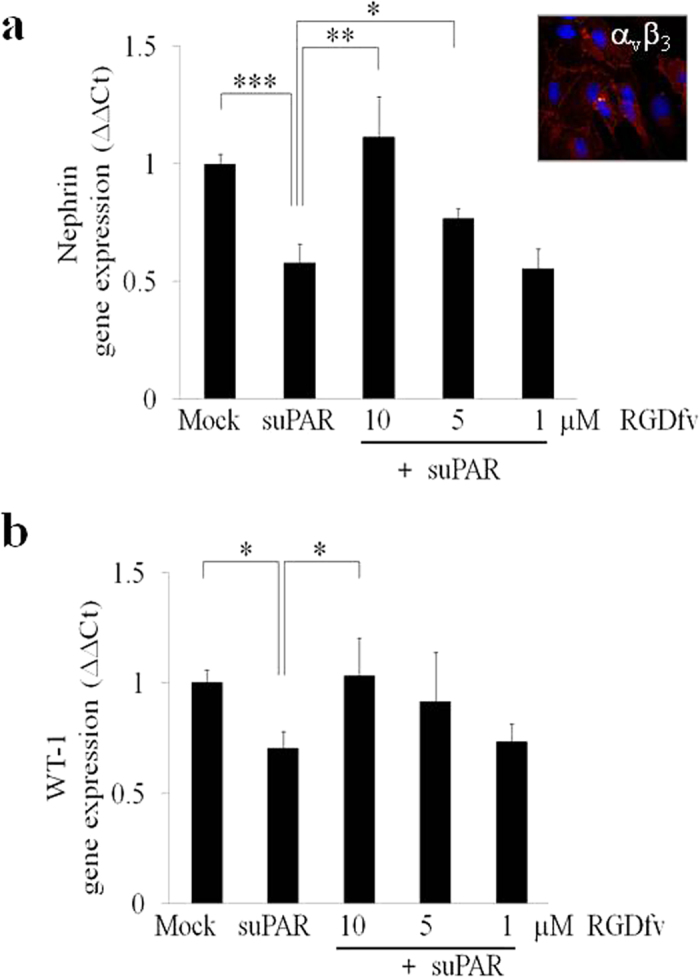
SuPAR mediated down-regulation of nephrin depends on α_v_β_3_ integrin interaction and is associated with reduced activity of WT-1. (**a–b**) Quantification of qPCR analysis of nephrin (**a**) and WT-1 (**b**) expression in Mock and suPAR treated (20 ng/mL) for 24 hours in CIHPs pre-incubated with different concentration (1, 5 and 10 μM) of αvβ3 antagonist (RGDfv). Results obtained by using the specify human TaqMan assays are expressed as relative fold change in treated cells vs. mock cells (ΔΔCt) and represent the average of 3 independent experiments ±SD. Values were normalized to the expression of GAPDH gene. Right upper picture of panel A shows one representative immunoflourescence staining out of 3 of αvβ3 (594 Alexa Fluor) integrin expression in red and nucleus in blue (DAPI) in CIHPs.

It has been demonstrated that suPAR binds and activates αvβ3 integrin in human podocytes^8^. In order to understand whether the αvβ3 integrin molecule is involved in suPAR dependent down-regulation of nephrin we used the αvβ3 small molecule-inhibitor cycloRGDfv^8^. Pre-treatment of CIHPs constitutively expressing αvβ3 integrin with cycloRGDfv prior stimulation with fl-suPAR resulted in a significant inhibition of suPAR-dependent downmodulation of nephrin at the transcription level (Fig. 4a). Moreover, qPCR analysis revealed that the effect of cycloRGDfv on nephrin expression on CIHPs incubated with suPAR is dose dependent, since incubation with 5 μM and 10 μM of this αvβ3 inhibitor, respectively, partially or fully restored the amount of nephrin transcripts compared to control experiments. These findings suggest that suPAR is able to induce the down-modulation of nephrin in CIHPs via αvβ3 interaction

**Figure 5:**
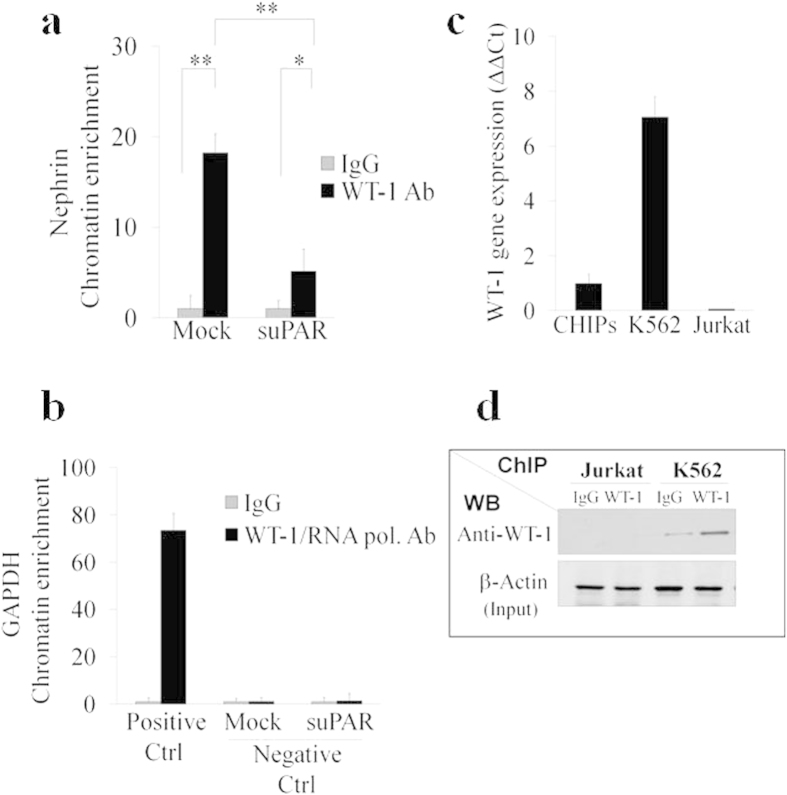
SuPAR induces lower WT-1 binding to the *Nephrin* gene promoter. (**a**) Chromatin immunoprecipitation (ChIP) analysis of WT-1 binding to the *cis* region of *Nphs1* gene promoter in CIHPs. Results are represented as fold change in the enrichment of precipitated chromatin fragments of *Nphs1* gene promoter with either anti-WT-1 Ab or Rabbit Normal IgG in non treated (Mock) or treated with suPAR cells. Precipitated products were amplified by qPCR by using SYBR Green assay and normalized to DNA lacking any WT-1 site, located in the promoter of GAPDH gene of Input. (**b**) Binding of the specific anti-RNA polymerase II (CTD4H8) Ab to the DNA fragment of *GAPDH* gene was used as the positive control (Ctrl). Amplification of *GAPDH* gene promoter in the IgG and WT-1 chromatin immunoprecipitated of Mock and su-PAR treated samples were used as a negative Ctrl. ChIP samples were analyzed in triplicate and represented the average of 3 independent experiments ±SD. (**c**) QPCR analysis of *WT-1* gene expression in Jurkat and K562 and CIHPs cell lines. TaqMan assays of 3 independent experiments ±SD. Values were normalized to the expression of GAPDH gene. (**d**) Detection of WT-1 protein by Western blotting assay (WB) after the chromatin immunoprecipitation with IgG control or anti-WT-1 antibodies in Jurkat and K562 cells. Analysis of β-actin of Inputs were used for normalization. One representative cropped blot out of two is shown. Statistical significance (*P*) is indicated by asterisks and is represented as: *<0.05; **<0.01; ***<0.001.

Different activating and suppressing transcription factors have been identified as being involved in the transcriptional regulation of *Nephrin* gene expression^36^,^37^,^38^. The most documented transcription factor involved in the regulation of *Nphs1* gene expression is WT-1. Knockout, transgenic, and siRNA analyses have demonstrated the importance of WT-1 at several stages of kidney development^39^,^40^,^41^. Moreover, expression of WT-1 continues in podocytes of adult kidneys and is required for physiological levels of nephrin expression. Consistent with this observation, it was demonstrated that nephrin expression in podocytes is lower in the kidneys of mice with reduced expression of WT-1^37^,^42^. In addition, both *in vitro* and *in vivo* functional approaches have shown that WT-1 can bind and activate the nephrin promoter and that this binding is essential for nephrin-specific expression *in vivo*^37^,^43^. Since our results showed suPAR-dependent down-regulation of nephrin at transcription level, we assessed whether WT-1 is involved in this process. QPCR analysis of WT-1 transcription showed a statistically significant decrease of WT-1 expression in CIHPs after treatment with fl-suPAR (Fig. 4b), thus indicating that WT-1 is a possible target of an activated suPAR-αvβ3 signaling down-stream pathway. In line with the kinetic observed for the down-modulation of nephrin (Fig. 4a), experiments performed with different concentrations of cycloRGDfv inhibitor revealed a full restoration of WT-1 transcripts after pre-treatment with 10 μM of cycloRGDfv (Fig. 4b), while we still could observe a lower but significant inhibition of nephrin incubating CIHPs with 5 μM of the αvβ3 inhibitor. To assess the specific suPAR-dependent attenuating role of WT-1 transcription factor in nephrin gene expression we evaluated the binding of WT-1 in the promoter region of *Nphs1* by chromatin immunoprecipitation assay (ChIP). We detected a significantly lower binding of WT-1 protein in the regulatory region of *Nphs1* gene in fl-suPAR treated cells compared to the non-stimulated podocytes (Fig. 5a). Amplification of GAPDH promoter was used as a positive control in CIHPs by immunoprecipitation of chromatin with anti-RNA polymerase II antibody (Fig. 5b). GAPDH promoter is lacking any WT-1 site thus as a negative control we amplified GAPDH promoter after chromatin immunoprecipitation with IgG or anti-WT-1 antibody in Mock and suPAR treated samples, however, we did not observe any significant changes (Fig. 5b). Specificity of the anti-WT-1 antibody used in ChIP assay was tested in the Jurkat and in K562 cell lines, known to be respectively negative and positive cells for WT-1 expression (Fig. 5c–d)^44^,^45^. These data strongly indicate that the suPAR-dependent down regulation of nephrin might occur through decreased transcription levels of WT-1 factor resulting in the attenuated binding to *Nphs1* gene promoter and thus lower transcription of nephrin. In addition, we analyzed the expression of the transcription regulator Snail that has recently been proposed as a repressor of *Nephrin* gene expression^38^. However, although we found detectable levels of Snail in CIHPs, we did not measure any increased amount of Snail after suPAR treatment (data not shown).

### High-dose of suPAR in knock out Plaur^−/−^ mice inhibits nephrin and WT-1 expression in podocytes and induces proteinuria.

**Figure 6:**
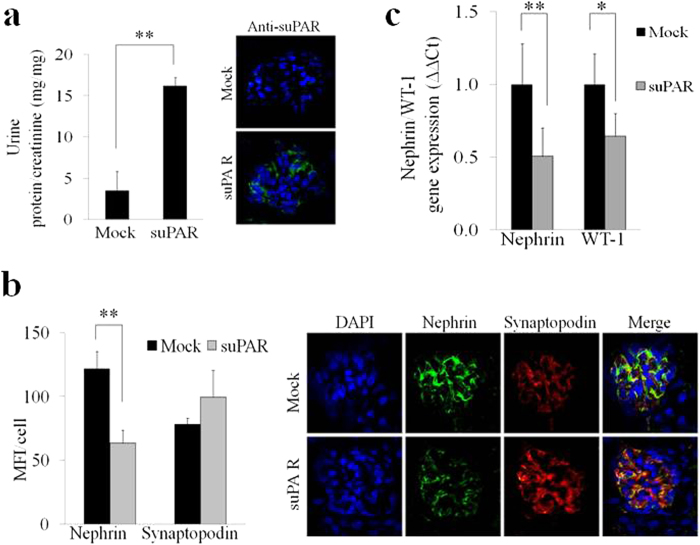
Injection of high doses of recombinant mouse suPAR into uPAR-knockout (Plaur^−/−^) mouse model induces down-regulation of nephrin expression. (**a**) Quantification (left panel) of the ratio between urine total protein (mg)/creatinine (mg) concentration of suPAR treated mice with high dose of 20 μg of mouse recombinant for 24 hours vs control mice (Mock) (N = 3 mice for group). Immune-fluorescence in green (right panel) of suPAR (488 Alexa Fluor) deposit into glomerular tissue of suPAR treated Plaur^−/−^ mice. (**b**) Quantification (left panel) of immunoflourescence staining of nephrin and synaptopodin expression in Mock and suPAR treated mice. (N = 3 mice for group). DAPI staining was used to determine cell numbers. Data are expressed as average of MFI/cell ±SD. Representative immunoflourescence staining (right panel) of nephrin in green (488 Alexa Fluor), synaptopodin in red (594 Alexa Fluor) and nucleus in blue (DAPI) expression in untreated (Mock) and suPAR treated mice (N = 3 mice for group). **(c)** QPCR analysis of nephrin and WT-1 expression in Mock and suPAR treated mice obtained by using specific mice TaqMan assays and expressed as relative fold change ±SD vs. mock cells. (N = 3 mice for group). Statistical significance (*P*) is indicated by asterisks and is represented as: *<0.05; **<0.01; ***<0.001.

We next examined whether exogenous circulating full-length suPAR could cause nephrin down-modulation in uPAR-knockout (Plaur^−/−^) mice in which we injected i.v. 20 μg (1 mg/Kg) of murine full-length recombinant mouse suPAR. After 24 hours, we observed an increased level of proteinuria and higher amount of suPAR deposit in the glomeruli of Plaur^−/−^ mice infused with full-length recombinant murine suPAR compared to control experiments (Fig. 6a)^8^. Moreover, we performed experiments using confocal microscopy in the same experimental setting and we observed a significant down-modulation of nephrin expression with no changes in synaptopodin levels in Plaur^−/−^ mice infused with full-length recombinant murine suPAR compared to control experiments (Fig. 6b). Finally, we confirmed our results obtained *in vitro* in the *in vivo* model by showing that the decreased expression of nephrin is associated with the down-modulation of WT-1 in suPAR treated Plaur^−/−^ mice (Fig. 6c). These data demonstrate that suPAR is able to activate a specific repressor-signaling pathway that leads to suppression of WT-1 and Nephrin genes.

## Discussion

Recently scientific opinion hailed the discovery of suPAR as a possible pathogenic factor as well as a mere biomarker of FSGS^8^. In addition different clinical studies observed increased suPAR concentration in various glomerular diseases thus implying on one hand the non specific role of suPAR in FSGS and on the other hand its emerging active pathological role in different glomerular and proteinuric unrelated to FSGS, disorders^8^,^9^,^10^,^11^,^12^,^13^. Indeed, in all studied renal disorders, increased suPAR was inversely associated to estimate glomerular filtration rate (eGFR) and in some reports to proteinuria. Nephritic syndromes represent characteristic clinical features also of other human diseases such as HIV and Hantavirus infection, diabetes and cardiovascular disorders that have been associated with increased level of suPAR^14^,^46^,^47^,^48^,^49^,^50^,^51^,^52^. Experimental studies both *in vitro* and *in vivo* clearly demonstrated the effect of suPAR on αvβ3 integrin activation in podocytes^8^,^33^. In addition, studies using the Plaur^−/−^ mice model confirmed the ability of high dose of suPAR to induce proteinuria^8^. Our study demonstrated that full length suPAR induced selective down-modulation of nephrin expression in human podocytes via interaction with αVβ3 integrin. This negative regulation of nephrin was observed both at the protein and the transcriptional levels, and was associated with a reduced level of the transcription factor WT-1. Furthermore, in the *in vivo* suPAR knock out Plaur^−/−^ mice model, the infusion of a high dose of suPAR correlates with lower expressions of nephrin and WT-1 in podocytes and glomerular permeability. Controversial results were obtained in wild type mice infused with high dose of suPAR^53^. These observations suggest that different molecular mechanism(s) may be involved in the detrimental action of suPAR in kidney physiopathology and various factors may control, inhibit or emphasize the toxic action of suPAR in pathological conditions. In this context, expression of αvβ3 integrin, which is expressed at low levels in podocytes under physiological conditions and increases in some pathologies, such as diabetic nephropathy, could play an important role^54^,^55^. Activation of αvβ3 integrin in podocytes can be also inhibited by other integrins such as α3β1 that represents the principal integrin expressed in podocytes and interacts with glomerular basement membrane^30^. On the other hand, an αvβ3 genetic polymorphism or other circulating factors such as TNF-α may affect suPAR activity^56^,^57^. Finally, the heterogeneity of circulating suPAR isoforms might explain why this biomarker, although being elevated in a variety of diseases, lacks disease-specificity and shows heterogeneous pathogenic action^2^. Besides the full length and cleaved form of suPAR various glycosylated variants of suPAR among different cell types have been reported^2^.Here, we show that only the full-length suPAR, but not c-suPAR, induces the down-modulation of nephrin, providing a conceptual framework for its pathogenetic action on podocytes in different human pathologies characterized by elevated suPAR and opening new therapeutic perspectives in the field. As an example, it might be interesting to evaluate the possible pathogenic role of suPAR in HIV infection since one of the clinical manifestation of kidney disorders in HIV pathogenesis is FSGS and increased levels of plasma suPAR in HIV patients have been correlated with disease progression^14^,^58^,^59^. Lymphoid organs of HIV infected individuals showed as an important site of production and release of suPAR and in particular full-length suPAR was found to be increased and contributes to prevent the anti-HIV activity of uPA^6^,^14^,^60^,^61^. In addition, we observed that plasma from HIV-infected individuals with increased levels of plasma suPAR have potential to induce downmodulation of nephrin (unpublished observation) and thus implicate suPAR as a possible renal risk factor in HIV pathogenesis.

## Methods

### Isolation and culture of human podocytes

Human renal tissue was obtained at the Department of Urology, Istituto Clinico Humanitas (Milan, Italy) from patients that underwent to both laparoscopic or open radical nephrectomy due to the renal cell carcinoma. All patients participated in this study provided written informed consent. All experimental protocols were approved by IRB (Authorization nr. 794/2011 Ethic Committee, Humanitas Clinical and Research Center, Milan). All methods were carried out in accordance with the approved guidelines. All tissues were collected from the distal area of the pathological tissue and macroscopically free from any disease as verified by haematoxylin-eosin-staining. Under aseptic conditions kidneys were minced into small pieces and then pressed through a series of stainless steel sieves (sieving method) with decreasing pore size of 200-μm, 100-μm and 75-μm. As a final step the glomeruli were collected on 75-μm sieve, washed twice and cultured in collagen IV (Sigma-Aldrich), coated plates in F-12 medium (Sigma-Aldrich) with 10% FBS, 2 mM ultra-glutamine, 100 U/mL penicillin, 100 μg/mL streptomycin, nonessential aminoacids (all purchased from Lonza Verviers Sprl) and supplemented with insulin-transferrin-sodium-selenite media supplement (100X, Sigma-Aldrich) and 0.35 ng/mL hydrocortisone (Sigma-Aldrich). After 3–4 days non attached glomeruli were washed and cultured for another 5–10 days. Conditionally immortalized human podocytes (CIHPs, kind gift from Dr. M. A. Saleem) were developed from primary human podocytes by transfection with the temperature-sensitive SV40-T and cultured as described in Saleem M.A. *et al.*^34^.

### Reagents

Human full length suPAR (composed of the three domains DI, DII and DIII) and truncated suPAR (c-suPAR, composed of the two domains DII and DIII) were purified as previously described^62^, and kindly provided by Dr. Massimo Resnati. RGDfv was purchased from Sigma-Aldrich (Saint Luis, MO, USA).

### QPCR

Total RNA was extracted using RNeasy mini columns (Qiagen, Valencia, CA), following manufacturer’s instructions, and its concentration was determined by spectrophotometry. One μg of total RNA was used to generate cDNA templates for RT-PCR, using random primers, RNase inhibitor and High-Capacity cDNA Reverse Transcription Kit from Applied Biosystem (Foster City, CA). All mouse and human gene expression were analyzed by the Taqman® mRNA specific assays for: nephrin, podocin, synaptopodin, WT-1, Snail and GAPDH (Applied Biosystem).

### Immunofluorescence

Human kidney tissue embedded and frozen in OCT or cells grown on coverslips coated with human collagen IV were fixed with 4% paraformaldehyde (PFA) then washed, and immunolabeled over night at 4 °C with rabbit polyclonal anti-nephrin (clone Y17-R, Acris Antibodies, San Diego, CA, USA) or rabbit polyclonal anti-αvβ3-integrin (clone 23C6, Santa Cruz) Ab. The bound antibody was stained with FITC/Alexa Fluor 594-conjugated goat anti-rabbit Ab respectively. Mouse kidney tissue embedded and frozen in OCT were fixed in acetone and stained for anti-nephrin (clone Y17-R) and subsequently with anti-synaptopodin (clone G1D4) followed by staining with FITC/Alexa Fluor 594-conjugated goat anti-rabbit and anti-mouse Ab respectively. SuPAR deposits in glomeruli were detected in frozen kidney tissue, fixed with 4% PFA and stained with antibody against murine uPAR (si420), kindly provided by Dr. Nicolai Sidenius (IFOM-IEO, Milan, Italy). Nuclei were stained with DAPI.

### suPAR knock out Plaur^−/−^ mice model

UPAR knock-out (Plaur^−/−^) mice^63^ were kindly provided by Dr. Nicolai Sidenius (IFOM-IEO, Milan, Italy) and maintained on C57BL6/N genetic background under specific pathogen-free conditions. Eight to ten week-old Plaur^−/−^ mice were intravenously injected with 20 μg (1 mg/Kg) of murine recombinant full length suPAR (R&D system). Twenty-four hours after injections, urine were collected and analyzed for creatinine and total protein content. Animals were then sacrificed and kidneys were collected and stored in OCT for immune fluorescence analyses and RNA extraction. Animal experiments adhered to the requirements of the Commission Directive 86/609/EEC and to the Italian legislation (Decreto Legislativo 116; 27 January 1992). All experimental protocols were approved by the Animal Care and Use Committee (Authorization nr. 192/2012-B, Humanitas Clinical and Research Center, Milan, Italy). All methods were carried out in accordance with the approved guidelines.

### Chromatin Immunoprecipitation

Chromatin immunoprecipitation (ChIP) assays were performed with the use of EZ-Magna ChIP Chromatin Immunoprecipitation Kit (Millipore) following manufacturer’s instructions. CIHPs were growth at 80–90% of confluency. Jurkat and K562 cell lines (ATCC) were cultured in RPMI 1640 medium supplemented with 10% FBS, 2 mM ultra-glutamine, 100 U/mL penicillin, 100 μg/mL streptomycin at concentration of 2 × 10^6^ cells/mL. Protein-DNA complexes were cross linked with 1% formaldehyde followed by glycine 0.125 M treatment, then cells were harvested and nuclear extraction was performed. Nuclei were collected by centrifugation at 12000 g and were suspended in sonication buffer with protease inhibitor cocktail and sheared to an average length of 750 bp by using Bioruptor Plus UCD-300 on high power for 36 cycles (30’’ ON and 30’’ OFF). Aliquots of cross-linked chromatin (50 uL) were diluted with 450 uL of ChIP dilution buffer and incubated overnight at 4 °C with 20 uL protein A/G magnetic beads and 5.0 μg/mL of rabbit polyclonal ChIP grade anti-WT-1 antibody (clone-C19, Santa Cruz Biotechnology). Mouse monoclonal anti-RNA polymerase II (CTD4H8) as the positive control was used and mouse/rabbit normal IgG were used as negative controls. 1% of non immunoprecipitated chromatin was saved as input sample. Cross-links between proteins and DNA were reversed by addition of ChIP elution buffer with proteinase K and incubation at 65 °C. DNA was purified using spin columns. Quantitative amplification of precipitated DNA fragments was performed on a 7900HT Fast Real-Time System (Applied Biosystem) using SYBR Green assay. The following primer pairs were used *Nphs1* promoter: 5′ CGCCCAGTCTCTTTATCTTTC–3′, 5′–GACAAGGAGCAGGAGTGAG– 3′; GAPDH promoter: 5′–TACTAGCGGTTTTACGGGCG–3′, 5′–CGAACAGGAGGAGCAGAGAGCGA-3′. The specificity of anti-WT-1 antibody used for ChIP assay was tested in Western blotting assay. All Inputs and chromatin immunoprecipitated samples with IgG or with anti-WT-1 rabbit polyclonal antibody (clone-C19, Santa Cruz Biotechnology) were separated by SDS-PAGE and transferred to nitrocellulose membrane. The membrane was blocked in 5% milk, incubated overnight at 4 °C with the goat polyclonal anti-WT-1 primary antibodies (Abcam, ab96792) or goat polyclonal anti-β-actin (clone C-11, Santa-Cruz Biotechnology, Santa Cruz, CA, USA), washed and incubated with secondary Ab -conjugated with horseradish peroxidase. Western blot analysis was conducted according to standard procedures using Immun-Star^TM^ WesternC^TM^ chemiluminescence detection substrate kit (Bio-Rad, Hercules, CA, USA).

### Statistical analysis

The significance of the data was assessed using ANOVA statistical analysis. Data shown are means ± S.D. The number of experiments is specified in the Figure legends. In the Figure statistical significance is indicated by asterisks (*). *P < 0.05; **P < 0.01; ***P < 0.001.

## Additional Information

**How to cite this article**: Alfano, M. *et al.* Full-length soluble urokinase plasminogen activator receptor down-modulates nephrin expression in podocytes. *Sci. Rep.* **5**, 13647; doi: 10.1038/srep13647 (2015).
